# Effects of Aging and Immersion on the Healing Property of Asphalt–Aggregate Interface and Relationship to the Healing Potential of Asphalt Mixture

**DOI:** 10.3390/ma16093574

**Published:** 2023-05-06

**Authors:** Haimei, Lili Li, Qinglin Guo, Tongmao Zhao, Pan Zuo, Fengming E

**Affiliations:** 1 Inner Mongolia Vocational and Technical College of Communications, Chifeng 024005, China; 2 School of Civil Engineering, Hebei University of Engineering, Handan 056038, China

**Keywords:** asphalt–aggregate interface, self-healing level, moisture and aging, healing potential of asphalt mixture, finite element simulation

## Abstract

The self-healing ability of asphalt–aggregate bonding interfaces can maintain the mechanical properties of asphalt mixtures. However, the interface’s healing ability will also be affected by moisture and aging. In order to clarify the influence of moisture and aging on the healing ability of a bonding interface, the effects of healing period and temperature on the self-healing level of interfacial strength were measured. The healing master curve of the strength was established. Thereafter, the effects of soaking time, salt solution concentration, and thermal aging on the healing degree of interfacial strength were measured. Based on digital image processing technology and the meso-finite element method, the influence of the interface on the healing performance of the mixture was simulated and analyzed, which was then verified by the beam bend healing test. The results show that the healing index of bonding strength increases with the ascent of healing temperature and period. Healing index gradually decreases with the extension of soaking period, and the higher the concentration of salt in the solution, the worse the healing performance of interfacial strength. After asphalt aging, the healing potential of the interface is weakened. There is a good linear relationship between the healing level of an asphalt–aggregate interface and the level of strength and fracture energy of the mixture. However, the actual healing level of an asphalt mixture is obviously lower than that of the interface, due to the addition of mineral filler. This paper provides a method for predicting the recovery performance of asphalt pavement.

## 1. Introduction

The self-healing ability of materials entails that the materials can heal the damage and cracks when micro-cracks and local damage occur inside of the materials, so that the material’s performance can be partially or fully recovered [1,2,3,4,5]. For asphalt pavement, cracks, pits, and other diseases may reduce the service life of the pavement and increase the maintenance cost. These diseases are mainly caused by the decay of asphalt adhesion, which is caused by asphalt aging, moisture damage, and other factors. Zhu et al. [1] found that the self-healing of asphalt at medium and high temperatures made the failed asphalt–aggregate interface adhere again, showing a self-healing characteristic of the interface, which makes an asphalt mixture with micro-cracks heal to a certain extent and recover its strength, and then prevents the cracks from expanding. Guo et al. [2] indicated that the strength and pre-peak failure energy of an asphalt mixture can be recovered to more than 80% after healing for 15 min at 90 °C. Therefore, the self-healing ability of asphalt concrete is helpful to improve the durability of pavement and prolong its service life [6,7,8,9,10,11].

In order to conveniently evaluate the adhesive strength between the asphalt and aggregate, the bitumen bond strength (BBS) test is gradually introduced to measure the adhesive property of an asphalt–aggregate interface [12]. Johannes et al. [13] carried out the bonding performance test of asphalt materials through a BBS experiment and evaluated the moisture damage resistance of asphalt in chip seal by using the bonding strength. Copeland et al. [14] investigated the influence of aging on asphalt adhesion through the BBS test. The correlation between the bonding strength of bitumen and rutting test results was determined. Hoki et al. [15] evaluated the effect of film thickness and soaking time on the bonding strength of interfaces using the BBS test. They indicated that the bonding strength declines with the increase in film thickness and soaking time. Guo et al. [16] studied the influence of water soaking, salt solution soaking, and freeze–thaw cycles on the bonding strength of the asphalt–aggregate interface by the BBS test, and established a coupled damage model considering soaking time, solution concentration, and freeze–thaw cycles to predict the bonding strength of an interface. The results showed that the BBS test can accurately measure the bonding strength between the asphalt and aggregate quickly, and reflect the bonding level between asphalt and the aggregate of an asphalt mixture.

In recent years, Hu et al. [17] studied the tensile strength of the bonding interface and self-healing ability of high-viscosity modified asphalt. Their results showed that the modifier reduced the self-healing ability of asphalt, and high-temperature immersion would lead to the decrease in adhesion and healing ability. A high-viscosity modifier enhances the polarity of modified asphalt and increases the thickness of structural asphalt by absorbing the light components, thus improving the bonding performance. Huang et al. [18] indicated that the moisture damage mechanism of asphalt pavement was mainly induced by the cohesion and adhesion damage of the asphalt–aggregate interface.

Sun et al. [7] indicated that the healing of asphalt–aggregate interface includes the cohesion healing and adhesion healing. Recently, Xu et al. [19] pointed out that asphalt aging caused the oxidation reaction, and the index of carbonyl and sulfoxide functional groups increased, which eventually led to a decrease in the self-healing performance of the binder. Zhou et al. [20] investigated the self-healing performance of five kinds of modified asphalt using the BBS test, and analyzed the influence of moisture and modifier on the interface’s healing ability. The results showed that the self-healing ability of different asphalt types was also different, and the void at the aggregate’s surface had a direct influence on the molecular selectivity and the healing ability of the interface. The self-healing process of the bonding interface was scanned using CT technology. Higher temperatures and dry conditions are beneficial to the self-healing of asphalt, while moisture is not conducive to the short-term or long-term healing of asphalt, but it can improve the healing rate at the middle stage [21,22]. In 2021, Huang et al. [23] carried out a bonding interface healing test and a four-point bending fatigue test, evaluated the healing performance of asphalt–aggregate bonding interfaces, and analyzed the correlation between the healing level of the interface and the fatigue healing performance of the mixture. Their results showed that there was a good linear correlation between the healing level of the bonding interface and the healing potential of the mixture.

Based on the above analysis and experimental investigation, it can be inferred that aging and moisture would affect the healing ability of the asphalt–aggregate interface, and the healing ability of the interface would influence the healing performance of the asphalt mixture. In fact, the pavement bears the actions of rainfall, high temperature, ultraviolet rays, and so on. The bonding strength and healing ability of the asphalt–aggregate interface will gradually decrease, which directly affects the self-healing ability of the mixture. However, the healing process of the asphalt mixture includes the adhesion healing of the interface and the cohesion healing inside of the asphalt mortar [24]. Although the current research has demonstrated the influence of single factors on the self-healing property of the interface, an understanding of the relationship between the healing level of the interface and the healing potential of the mixture was necessary to improve the mixture durability. It is difficult to quantitatively analyze this relationship solely through experiments. Fortunately, finite element simulation technology provides a convenient way to explore this relationship. 

**Figure 1:**
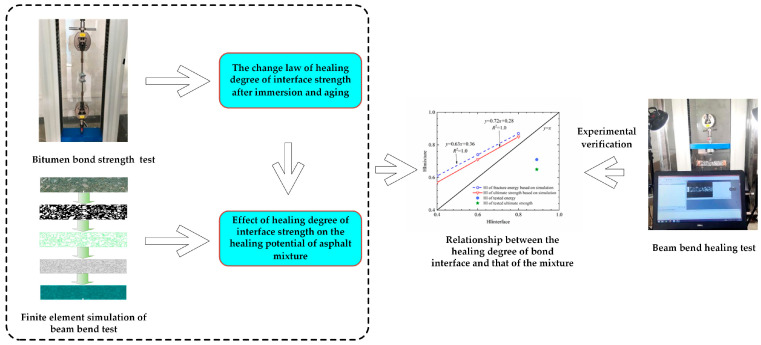
Research algorithm.

The research goal of this work is to determine the influence law of soaking and aging on the healing ability of the asphalt–aggregate interface, and to explore the relationship between the interface’s healing ability and the recovery degree of the mixture’s performance. In view of this goal, the relationships among healing time, healing temperature, and healing index of the asphalt–aggregate interface were measured first in this paper. Based on the principle of time–temperature equivalence, the self-healing master curve of the interfacial strength is established, and the effects of different factors, including soaking time, salt solution concentration, and aging degree, on the healing characteristics of the asphalt–aggregate interface are investigated. The finite element simulation was carried out in order to evaluate the healing performance of the asphalt mixture. The influence of the healing ability of the asphalt–aggregate interface on the self-healing potential of the asphalt mixture was analyzed and verified. The research algorithm is described in Figure 1.

## 2. Materials and Methods

### 2.1. Materials

**Figure 2:**
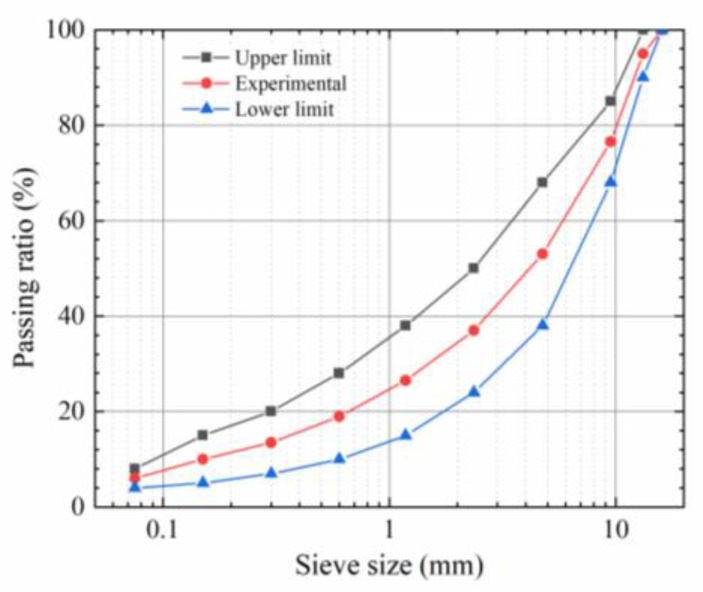
Experimental gradation.

The asphalt used in this experiment was AH-70# petroleum asphalt, which has a penetration grade of 70. Its basic properties are presented in Table 1. Basalt aggregates were selected to prepare the asphalt mixture, and the apparent gravity and moisture uptake ratio of the aggregates are listed in Table 2. The gradation named AC-13, which is recommended by China technical specifications JTG D50-2017 [25], was selected for the experiment, as presented in Figure 2. According to the Marshall test results, the optimal asphalt content of the mixture was 5.0%, the apparent gravity of the mixture was 2.447 g/cm^3^, and the air void in the asphalt mixture was 4.1%. A plane specimen with a size of 30 cm × 30 cm × 5 cm was prepared to cut into the beams of 30 cm × 5 cm × 5 cm, and a notch of 5 mm in depth and 3 mm in width was cut at the bottom of the mid-span of the beam.

### 2.2. Healing Test of Asphalt–Aggregate Bonding Interface

**Figure 3:**
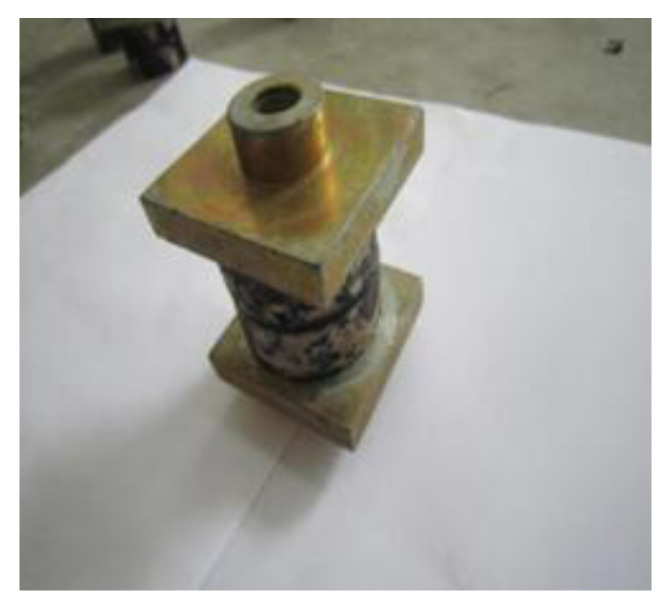
Sandwich specimen of the asphalt–aggregate bonding interface.

**Figure 4:**
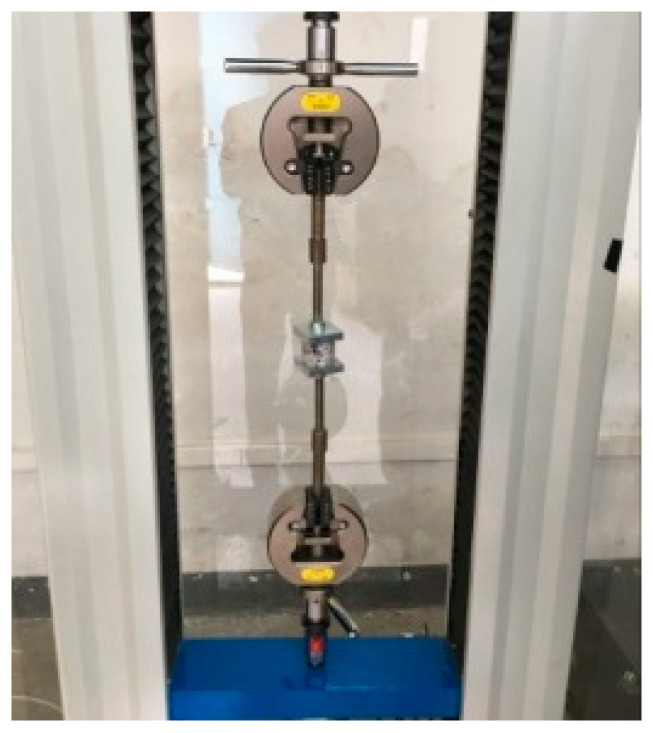
Bitumen bond strength (BBS) test.

A disc specimen, with a diameter of 4.2 cm, was prepared by coring the stone plate with a water drill, and its surface was polished with sandpaper to ensure that the roughness of the interface was consistent. The moisture inside of the disc specimen was removed by drying at 105 °C for 12 h. Thereafter, the disc specimen was placed in an oven at 135 °C for 4 h, taken out, and asphalt was dropped onto the center of the disc specimen, covered with another disc specimen, and compacted. The asphalt was required to evenly coat the specimen interface, and the thickness of the asphalt film was controlled at 0.1 mm. Based on the specific test method of reference [16], the “aggregate–asphalt–aggregate” bonded sample was finally made, as presented in Figure 3. Thereafter, the sample was cooled at room temperature, and the tensile test was carried out after it was kept at 20 °C for 6 h. In this paper, the electronic universal testing machine from cangzhou city of hebei province was used for the pull-off test, with a loading rate of 1 mm/min, and the test was stopped when the specimen was destroyed. Three parallel samples were set for each group test. The specific process is presented in Figure 4.

After the test, the damaged specimens were stacked and healed at 10 °C, 40 °C, 60 °C, and 80 °C. After the healing, the second pull-off test was carried out, and four parallel specimens were prepared for each group. The healing index of the interfacial strength was obtained in order to evaluate the healing performance of the interface. The healing index can be calculated using the following equation.

(1) $$HI=\frac{\sigma_{2}}{\sigma_{1}}$$

where, *HI* is the healing index of the interfacial strength; $\sigma_{1}$ and $\sigma_{2}$ are the tensile strengths before and after healing, respectively, MPa.

In seasonally frozen areas, snow-melting agents are often used to remove the snow. In order to investigate the influence of salt solutions on the self-healing performance of the interface, a salt solution was prepared by using Cacl_2_ to simulate the influence of salt corrosion on the healing performance of the interface. The asphalt was also aged using the rolling thin film oven test (RTFOT) method, and the influence of aging degree on the interface’s healing performance was discussed.

### 2.3. Beam Bend Healing Test of Asphalt Mixture 

**Figure 5:**
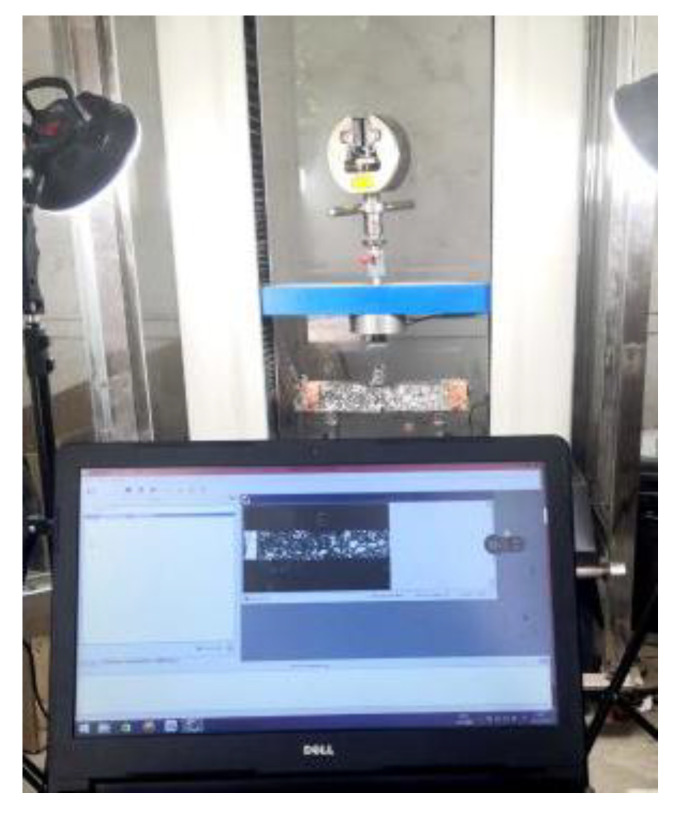
Three-point bending test.

**Figure 6:**
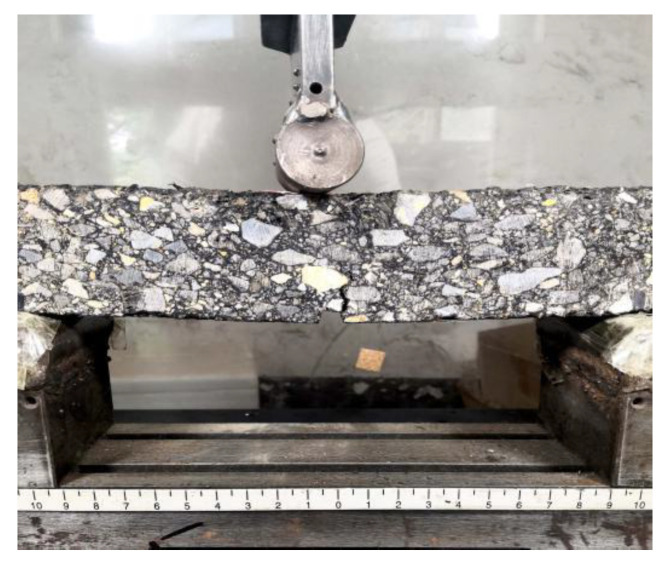
Bending failure of the beam specimen.

**Figure 7:**
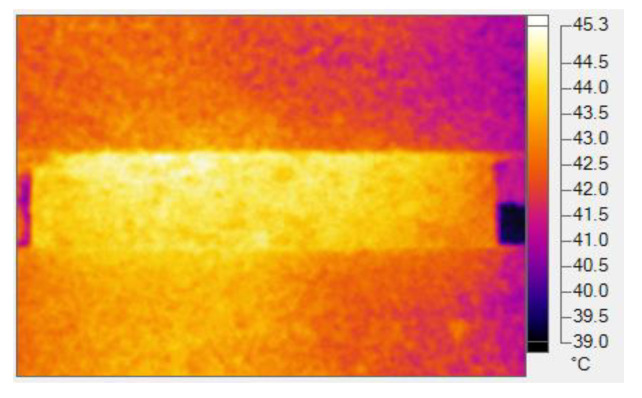
Temperature field of the beam specimen at 14:00 on 25 June 2019.

The healing of the bonding interface leads to the mechanical performance recovery of the asphalt mixture. In order to determine the self-healing ability of the asphalt mixture, the healing performance of the asphalt mixture in an outdoor, high-temperature environment was measured using the beam bend healing test. The bending test was carried out using the aforementioned beam specimen, and the support space was 200 mm ± 0.5 mm. The beam specimen was kept at 10 °C for more than 4 h, and then the bending failure test was carried out. The vertical loading rate was 1 mm/min. During the test, the deformation and load on the bottom of the mid-span were recorded in real time. The experimental procedure is presented in Figure 5 and Figure 6. In the first bend test, the test was stopped when the mid-span vertical deformation reached 2 mm. Subsequently, the damaged beam was placed outdoors for 48 h of healing. The healing temperature of the beam specimen was between 23 °C and 45 °C, with an average temperature of 28 °C. The typical temperature field on the beam’s surface is presented in Figure 7. After 48 h of healing, the second bending test was carried out after beam conditioning at 10 °C for 4 h, and the test procedure was consistent with the first bending test. Three parallel samples were set for each group test.

After finishing the test, the healing degree of the mixture was evaluated using the fracture strength and fracture energy. It can be determined according to the following equations.

(2) $$\sigma (t)=\frac{3LF(t)}{2bh^{2}}$$

(3) $$\varepsilon (t)=\frac{6td(t)}{L^{2}}$$

(4) $$G_{F}=\int_{0}^{\varepsilon f}\sigma \varepsilon d\varepsilon$$

(5) $$HI_{S}=\frac{R_{healed}}{R_{0}}$$

(6) $$HI_{GF}=\frac{G_{Fhealed}}{G_{F0}}$$

where, σ(t) represents the tensile stress at the bottom of the mid-span, MPa. ε(t) is the tensile strain at the bottom of the mid-span. $\varepsilon_{f}$ is the failure strain corresponding to 2 mm vertical deformation, F(t) is the applied load, N; *L* represents the support space, mm. *b* and *h* are the width and height of the beam, respectively, mm. d(t) is the deformation at the mid-span of the beam, mm. $HI_{s}$ is the strength recovery index, $R_{0}$ and $R_{healed}$ are the failure strength before and after healing, respectively, MPa. $G_{0}$ and $G_{Fhealed}$ are the failure energy before and after healing, respectively, J. $HI_{GF}$ indicates the healing index of failure energy.

### 2.4. Mesoscopic Finite Element Simulation for Asphalt Mixture

**Figure 8:**
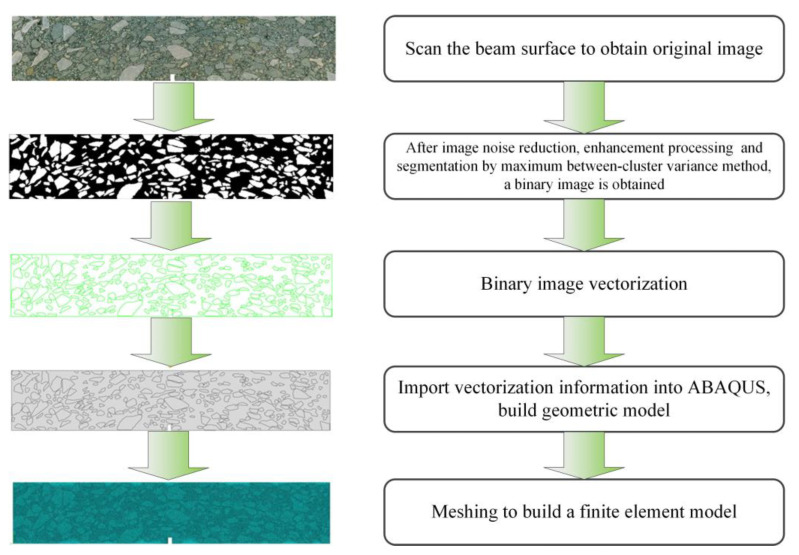
Reconstruction process of the 2D finite element model for the asphalt mixture.

The healing of the asphalt–aggregate interface will directly affect the healing performance of the mixture. However, the beam bend healing test can only analyze the self-healing ability of the mixture from the macroscopic perspective, and cannot establish the relationship between the interface healing level and the self-healing potential of the mixture. Herein, the meso-finite element method was adopted in order to reveal this relationship. A finite element (FE) beam model of the asphalt mixture was established, based on digital image processing technology, in order to consider the realistic mesostructure of the asphalt mixture. The modeling procedure is presented in Figure 8.

**Figure 9:**
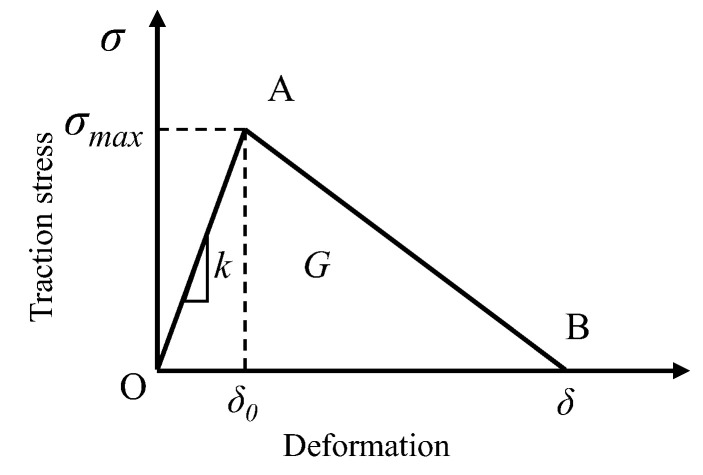
Tractor-separation curve of the CZM. Where *σ_max_* represents the maximum stress, MPa; *δ*_0_ is the deformation of the CZM element when the ultimate strength is reached. *k* represents the stiffness, MPa; G represents the failure energy of the CZM.

The cohesive zone model (CZM) has been widely used to investigate the interface debonding process in the material fracture field. Therefore, the bilinear cohesive zone model was selected in order to describe the constitutive relation of the bonding interface. The tractor-separation curve is presented in Figure 9.

**Figure 10:**
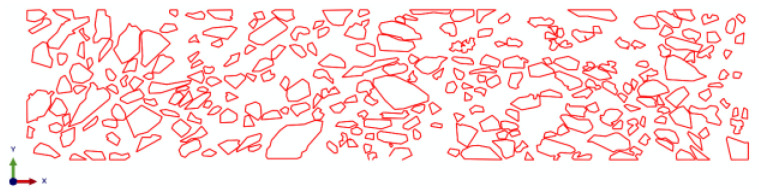
CZM elements between the aggregate and asphalt mortar.

**Figure 11:**
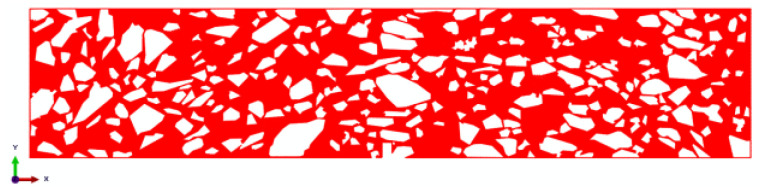
CZM elements inside of the asphalt mortar.

When the traction stress satisfies the following relationship, the cohesive element begins to be damaged.

(7) $${\left\{\frac{\langle T_{n}\rangle }{T_{n}^{0}}\right\}}^{2}+{\left\{\frac{T_{s}}{T_{s}^{0}}\right\}}^{2}=1$$

where < and > are Macaulay brackets, which indicates that the compression stress will not cause damage to the cohesive element. $T_{n}^{0}$ and $T_{s}^{0}$ are traction stresses in normal direction and shear direction, respectively. $T_{n}$ and $T_{s}$ are pure normal traction and pure shear traction, respectively, at the beginning of damage. Once the failure starts, the material goes into a softening state. This process is quantified by defining a damage variable *D*, as presented in Equation (8).

(8) $$D=\frac{\delta_{f}(\delta_{\max}-\delta_{0})}{\delta_{\max}(\delta_{f}-\delta_{0})}$$

where $\delta_{0}$, $\delta_{\max}$, and $\delta_{f}$ are the effective displacement at the beginning of damage, the maximum effective displacement obtained during loading, and the effective displacement at the point of complete failure, respectively. In this paper, the power-law failure criterion based on energy is used to describe the fracture evolution in mixed mode:

(9) $${\left\{\frac{G_{n}}{G_{n}^{c}}\right\}}^{2}+{\left\{\frac{G_{t}}{G_{t}^{c}}\right\}}^{2}=1$$

where $G_{n}^{c}$ and $G_{t}^{c}$ are the fracture energy in normal and shear directions, respectively. $G_{n}$ and $G_{t}$ are the dissipated energy generated in the normal and shear direction, respectively, during the loading procedure. In the meshing stage, the three-node plane stress element, called CPS3, was applied to mesh the asphalt mortar, and the four-node quadrilateral linear plane stress element, called CPS4R, was used to mesh the coarse aggregate. The FE model was discretized by free mesh, and the side length of the element was 0.5 mm. A self-compiled program developed by MATLAB was adopted in order to edit the initial ‘inp file’ generated by ABAQUS. In this process, the zero-thickness CZM element was inserted into the mortar and the interface, one after the other. The completed CZM model is presented in Figure 10 and Figure 11.

**Figure 12:**
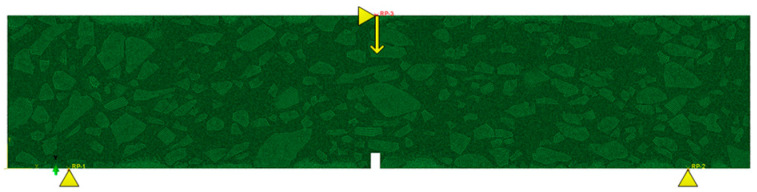
Model boundary and loading conditions.

The boundary and loading points of the beam are presented in Figure 12. The support spacing of the finite element model was 20 cm. The parameters of the CZM element, asphalt mortar, and aggregate are listed in Table 3, Table 4 and Table 5. The parameters in Table 3 and Table 4, from the results of Liu [26], were adopted as the initial values, and then the trial calculation was conducted. The final values were determined when the difference between the experimental and the simulated load-displacement curve before the peak was at its minimum. The parameters in Table 5 are consistent with the results of E [27].

## 3. Results and Discussion

### 3.1. Effects of Healing Period and Temperature on Healing Level of Interface Strength

**Figure 13:**
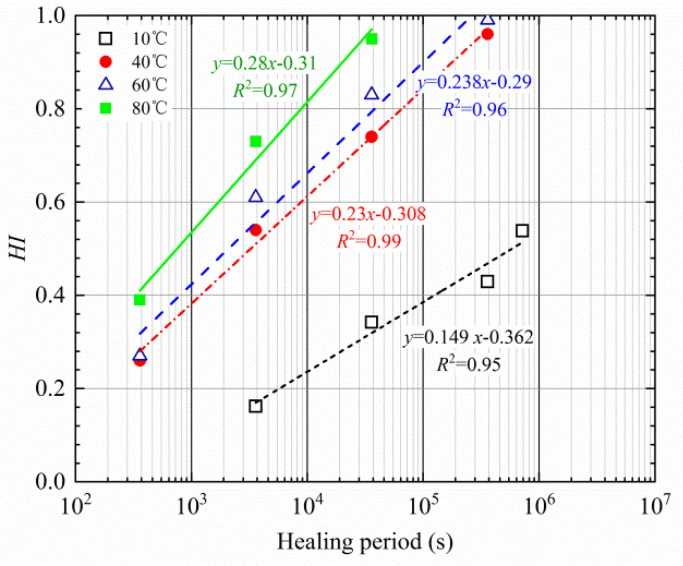
Healing-index of the bonding interface of asphalt–aggregate at different temperatures.

The healing level of the interface was measured using the bitumen bond strength test at different temperatures and healing periods, and the healing index of the bonding interface was calculated based on the tensile strength, as presented in Figure 13. 

As presented in Figure 13, the healing index of the bonding interface gradually increases with the extension of healing time. The higher the healing temperature is, the shorter the recovery period of the strength will be. For the same healing period, the higher the healing temperature is, the better the recovery of the strength is. The self-healing degree of the interface strength between the asphalt and aggregate obviously depends on the healing time and temperature. In order to obtain a good healing effect, it is feasible to prolong the healing time or increase the temperature. This is mainly because asphalt is a typical viscoelastic material. The higher the temperature is, the lower the viscosity of the asphalt, and the more active its molecular movement is. The accelerated molecular movement is helpful to enhance the healing of the asphalt film at the interface.

### 3.2. Master Curve of Healing Index

In this work, the Compertz model, as presented in Equation (10), was selected to describe the trend of the healing index, and the parameters of the healing model can be determined through the nonlinear least square method on the data in Figure 13.

(10) HI(t) = a exp(b exp(c × lgt))

where *a*, *b*, and *c* are model parameters. Referring to the change in *HI*, the parameters should follow the conditions: *b* < 0, *c* < 0; the parameters of the *HI* model are listed in Table 6. 

It can be observed from Table 6 that the correlation coefficients (*R*^2^) of nonlinear fitting are all above 0.96, which indicates that the Compertz model has good applicability, and it can accurately describe the change law of the healing index. A low RMSE indicates that there is little difference between the predicted value and the measured value. According to the development trend of the healing index in Figure 12, the healing index is influenced by both temperature and healing time. When the temperature is below 60 °C, the healing period is in the range of 100 s–1,000,000 s, while when the temperature reaches 80 °C, the healing time is sharply shortened, and when the healing time is 30,000 s, the healing index is close to 1. Thus, both *b* and *c* first increase and then decrease, which is caused by the cross influence of temperature and healing time.

In order to evaluate the interface healing ability at different temperatures, the temperature shift factor is calculated based on the reference temperature of 60 °C. A trial method is proposed to determine the initial value of the temperature shift factor considering the prediction accuracy of the master curve. On this basis, the glass transition temperature *T_g_* and the reference temperature *T_S_* can be determined by Equation (11).

(11) $$lg\alpha_{T}=\frac{-C_{1}\times (T-T_{S})}{C_{2}+T-T_{S}}$$

where *C*_1_ = 8.86 and *C*_2_ = 101.6.

There is a conversion relationship between the reference temperature and the glass transition temperature, as calculated by Equation (12).

(12) $$T_{S}=T_{g}+50$$

Generally, the temperature selected in the experiment is fixed. These temperatures may not cover *T_S_*. *T_S_* is unknown. Herein, a certain temperature *T*_0_ in the actual test sequence is often selected as the reference temperature, and the healing index at other temperatures can be moved to the curve of *T*_0_ after shifting from the test temperature to the reference temperature. The temperature shift factor can be determined using Equation (13).

(13) $$lg\alpha_{T0}=\frac{-8.86\times (T_{0}-T_{S})}{101.6+T_{0}-T_{S}}$$

The shift factor of any temperature to the reference temperature, defined as $lg\alpha \prime_{T0}$, can be obtained by using Equations (11) and (13).

(14) $$lg\alpha \prime_{T0}=lg\alpha_{T}-lg\alpha_{T0}=-C_{1}C_{2}\frac{-(T-T_{0})}{\left(C_{2}+T-T_{S})(C_{2}+T_{0}-T_{S}\right)}$$

According to the measured data of different temperatures, $lg\alpha \prime_{T0}$ can be determined by moving to the reference temperature, with the fixed reference temperature *T*_0_ at 60 °C, so that the initial value of the temperature shift factor relative to the reference temperature can be calculated by Equation (14) when $T_{0}=T_{S}$. Thereafter, the temperature shift factor is optimized by trial calculation, and SSE, *R*^2^, and RMSE are used to determine the optimal temperature shift factor with a minimum error. The results are presented in Table 7.

**Figure 14:**
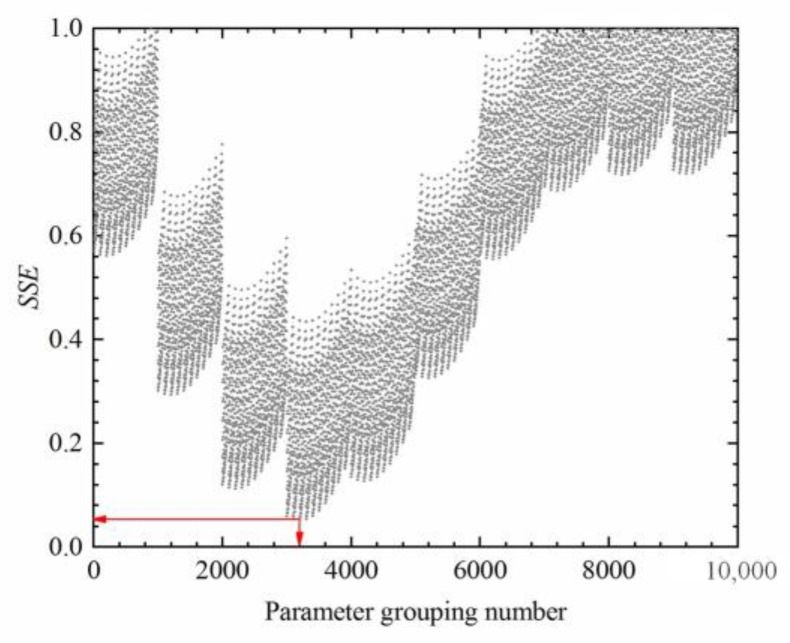
Variation of SSE in temperature shift factor optimization.

Based on the optimal value of $lg\alpha \prime_{T0}$ in Table 7, *T_S_* is 323.288 K and *T_g_* is 273.288 K. The temperature shift factor $lg\alpha_{T}$ can be determined according to Equation (11), which is listed in Table 8. Subsequently, the correlation coefficients *R*^2^ and SSE are used as control indexes to optimize $lg\alpha_{T}$ through the trial calculation. In the optimization process, the change in SSE is presented in Figure 14. The closer SSE is to zero, the smaller the difference between the predicted and the experimental *HI* is. The optimized results are also presented in Table 8.

**Figure 15:**
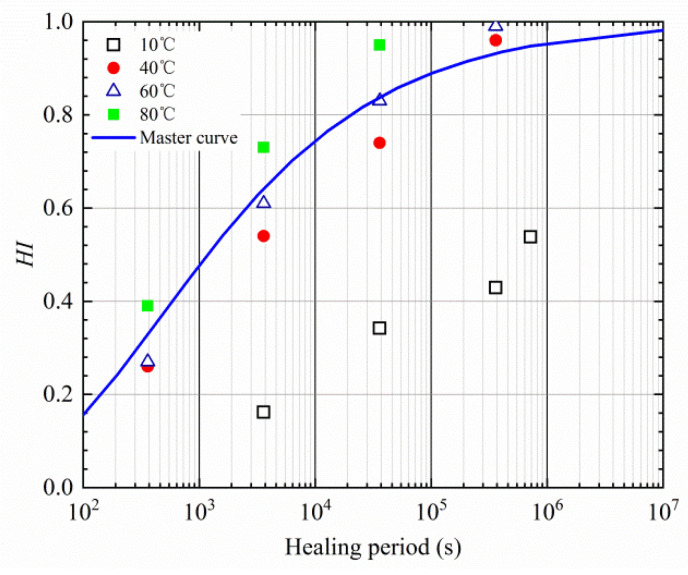
Master curve of *HI* based on the measured healing index.

The measured healing indexes of different temperatures were nonlinearly fitted based on temperature shift factor, as presented in Figure 15. Parameters of the master curve of the healing index are presented in Table 9.

### 3.3. Effects of Immersion and Aging on the Healing Level of Interface

**Figure 16:**
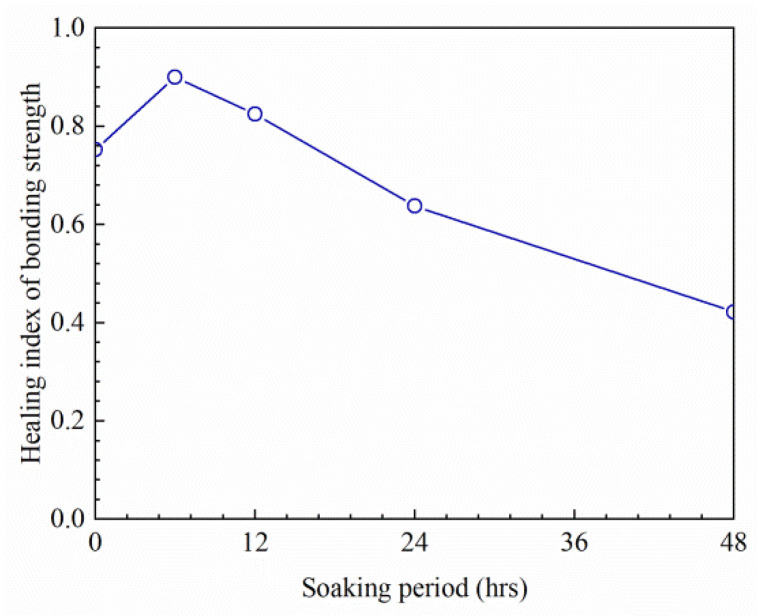
Influence of soaking period on the healing index of bonding strength.

Asphalt pavement will be affected by environmental factors, such as rainfall and aging, in the service stage. The change in asphalt molecular structure will inevitably change the healing level of the asphalt–aggregate interface. After soaking in water, the healing index of the bonding strength of the interface is presented in Figure 16.

As can be observed from Figure 16, the healing index of interfacial bonding strength is higher than those of untreated specimens when the soaking period is shorter than 16.8 h. This is because the immersion time is short, and a small amount of water enters the bonding area of the interface [8]. In addition, the specimen is soaked at 20 °C, which accelerates the heat transfer and promotes the healing of interface bonding. Therefore, the healing degree of bonding strength after short-term soaking is higher than those of untreated ones. After soaking for more than 6 h, the healing index of the strength decreases gradually. After 48 h of soaking, the healing index decreased by 34%. This proved that long-term soaking was unfavorable to the healing potential of the interface. The longer the soaking time is, the worse the self-healing performance of the interface is. With the extension of soaking time, water permeates into the interface to form a water film, which hinders the diffusion and re-bonding of asphalt molecules on the surface of the aggregate, resulting in a decline in self-healing level. It can be inferred that moisture has a significant effect on the healing ability of the asphalt–aggregate bonding interface. Zhou et al. [22] stated that moisture is not conducive to the short-term and long-term healing of asphalt, but it can improve the healing rate at the middle stage. The findings in this paper are consistent with those of Zhou. The middle stage is between approximately 6 and 12 h. Long-term water intrusion will reduce the self-healing potential of asphalt pavement, and eventually accelerate damage and cracking.

**Figure 17:**
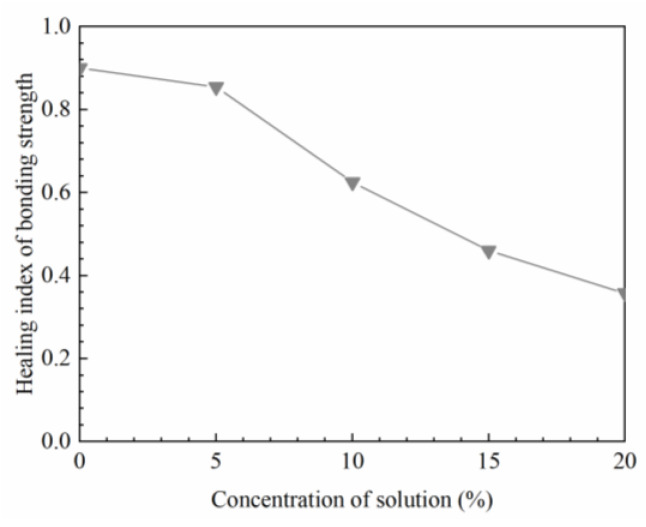
Influence of salt solution on the healing index of bonding strength after 6 h soaking.

In this test, the soaking time was set at 6 h. The change in the healing index is presented in Figure 17.

According to the changing trend in Figure 17, the healing index of interfacial bonding strength decreases with the increase in solution concentration. The self-healing property declines slowly when the concentration is no more than 5%. Afterwards, the healing index decreases linearly with the increase in the concentration. Therefore, a high concentration solution has a negative effect on the healing ability of the interfacial strength. Chlorine is the main component of snow-melting agents. It is necessary to control the dosage of chlorine salt when spraying snow-melting agents in winter, which is beneficial to reduce the concentration of salt solution and the influence of chlorine salt on the healing performance of asphalt pavement.

**Figure 18:**
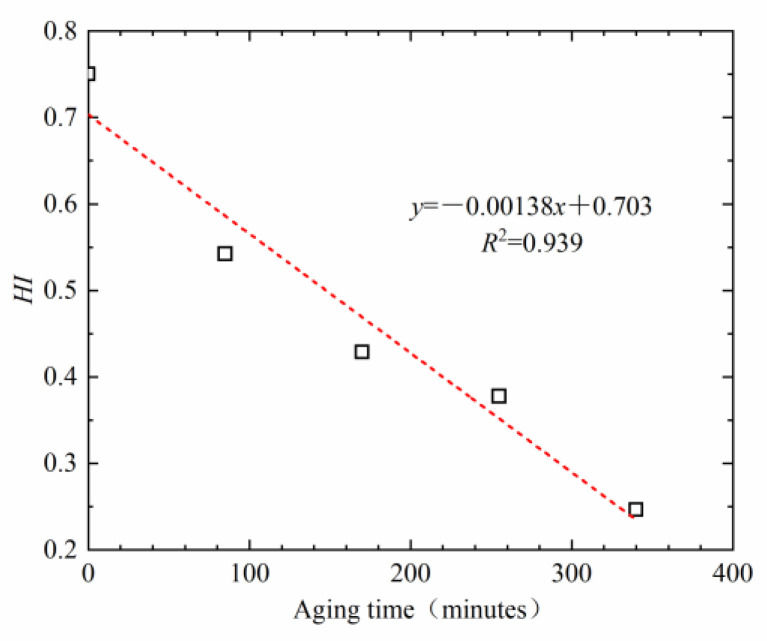
Influence of aging on the healing index.

In order to explore the influence of asphalt aging on the healing performance of the asphalt–aggregate interface, the asphalt was aged using the RTFOT method in a laboratory setting. The aged asphalt was adopted to make sandwich bonding specimens. The self-healing index of the asphalt–aggregate interface was then tested, as presented in Figure 18.

**Figure 19:**
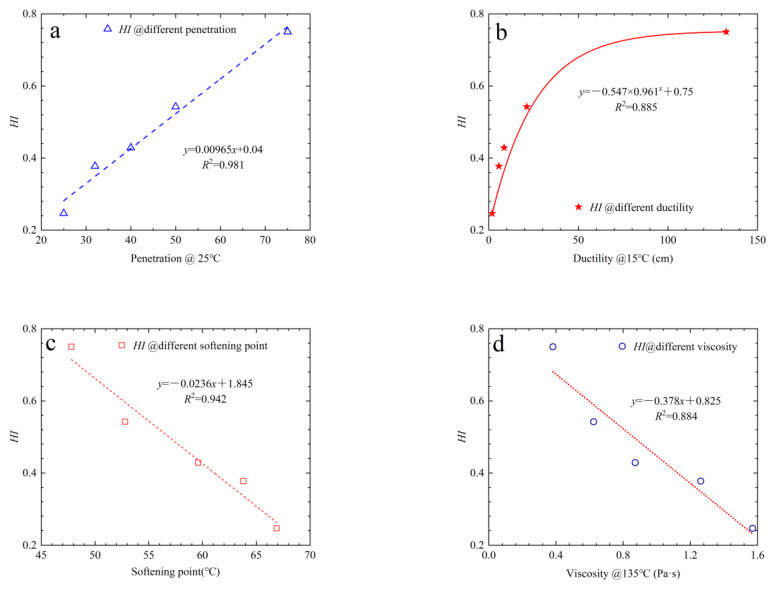
Relationship between the indexes of aged asphalt and the healing index. (**a**) *HI* @ different penetration, (**b**) *HI* @ different ductility, (**c**) *HI* @ different softening point, (**d**) *HI* @ different viscosity.

It can be observed from Figure 18 that with the prolongation of aging time, the healing index decreases linearly. The longer the time over which the asphalt is aged, the lower its healing index is, and the worse its healing ability is. This is because asphalt includes saturate, aromatic, resin, and asphaltene components. During the aging process, with the continuous input of oxygen, light components are gradually oxidized and the small molecules condense into asphaltene in asphalt, while aromatic and saturated components decrease, resulting in the change in asphalt colloid structure from a solution gel to a gel structure. On the other hand, the molecular chain of asphalt is destroyed and reorganized in the process of oxidation, which increases its relative molecular weight, as well as the movement resistance of asphalt molecules, and slows down the diffusion rate of asphalt molecules. Finally, the interface’s healing ability decreases. In addition, the penetration, ductility, softening point, and viscosity of asphalt before and after aging were also tested, and the correlation between healing index and these indexes was analyzed, as presented in Figure 19.

It can be observed from Figure 19a,b that the healing index rises with the increase in penetration. The healing index first rises rapidly with the ascent of ductility, and then the growth gradually slows down. It can be observed from Figure 19c,d that the healing index decreases linearly with the increase in softening point and viscosity. As a result, the aging of asphalt leads to the decrease in viscosity, the increase in softening point, and the deterioration of fluidity. However, there is a close relationship between the asphalt’s fluidity and the healing ability of the interface. Asphalt with a high penetration, low softening point, and high ductility has excellent healing ability. The changes in these indexes have macroscopic effects, which are caused by the change in asphalt components and colloid structure. For this reason, these common indexes may be used as an indirect basis on which to evaluate the healing potential of the bonding strength at the interface. It must be indicated that polymer-modified asphalts still require more data to support this statement in their case.

### 3.4. Relationship between the Interface Healing Level and Healing Potential of Mixture

**Figure 20:**
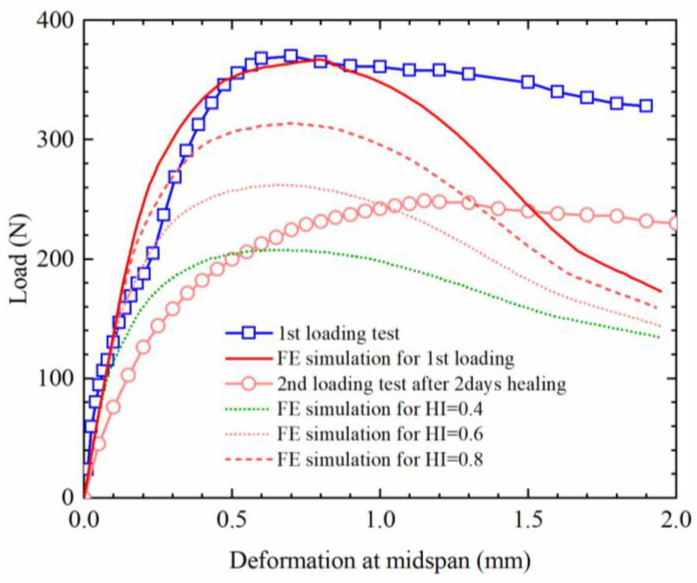
Load-displacement curves of the beam before and after healing.

To reveal the relationship between the healing index of the interface and the healing performance of the asphalt mixture, the self-healing performance of the asphalt mixture was simulated using the meso-finite element method in this paper, and the parameters for the cohesive zone model of the bonding element were designated to different healing levels, as presented in Table 10. The load-displacement curve of the bending beam for the asphalt mixture was obtained after finite element simulation and presented in Figure 19. The measured load-displacement curves are also demonstrated in Figure 20.

As described in Figure 20, for the first loading condition, the flexural bearing capacity of the beam is the highest, and the FE simulated and measured values before the peak are consistent. However, the simulated values begin to deviate from the measured values, and the load of the FE model degrades faster, while the actual asphalt mixture has good post-peak bearing capacity. This may be due to the fact that the CZM element did not consider the influence of asphalt viscosity. Although there are some differences between the experimental and simulated values, the meso-finite element method is still feasible in the simulation of the mechanical properties of the asphalt mixture. The finite element simulation results also show that the flexural bearing capacity of the healed asphalt mixture gradually declines with the decrease in the healing index of the interface. Comparing the measured and simulated results of the asphalt mixture, it can be observed that the flexural bearing capacity of the asphalt mixture is very close to the condition that the healing index of the interface is 0.6 when the beam specimens of the asphalt mixture were healed at 23–45 °C (the average temperature was 28 °C) for 2 days. It is helpful to save the waiting time for the healing process, speed up the analysis procedure, and save the test consumption.

**Figure 21:**
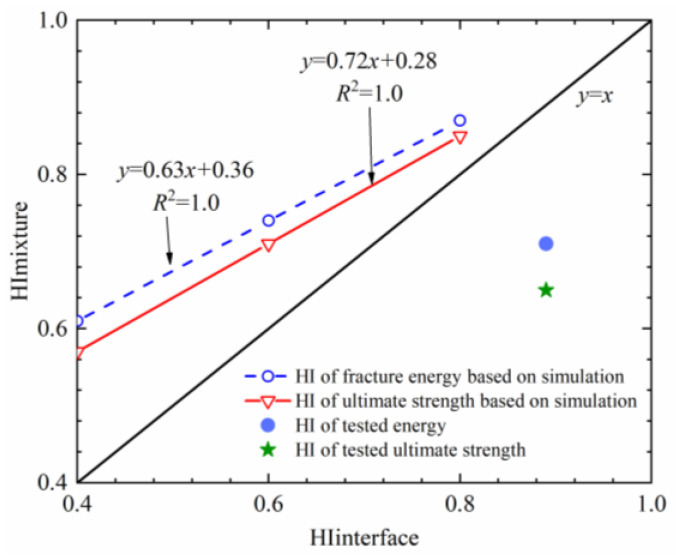
Healing level between the interface and the mixture.

In order to clarify the relationship between the healing performance of the interface and the healing potential of the mixture, the healing performance of the interface and the healing degree of fracture energy or ultimate strength of the mixture were compared, as presented in Figure 21. In this paper, the average healing temperature of the mixture is 28 °C, and the *HI* of the interface after healing can be calculated by using the master curve. 

As presented in Figure 21, it can be observed that there is an obvious linear correlation between the healing index of the bonding strength of the asphalt–aggregate interface and the self-healing performance of the mixture. It can be observed from this relationship that the self-healing ability of the asphalt–aggregate interface has a direct impact on the performance of the asphalt mixture. On the basis of the simulated and the measured results, the energy recovery of the asphalt mixture after self-healing is the fastest, and the self-healing amplitude of the strength is slightly lower than the fracture energy. It is proved that there are significant differences among different indexes for evaluating the healing potential of the asphalt mixture. The simulation results show that the healing index of the asphalt mixture will be higher than that of the interface, while the measured results show that the healing level of the mixture (0.65–0.71) is obviously lower than that of the interface (0.89). This is mainly because the interface studied in this paper only includes asphalt and stone, without considering the influence of mineral powder and fillers. In addition, the bilinear failure criterion of the CZM element and 2D FE simulation also result in a difference between the test value and the simulation value. Cheng et al. [28] and Varma et al. [29] stated that mineral powder would increase asphalt viscosity. According to the relationship in Figure 19d, the increase in viscosity leads to a decrease in the self-healing ability of the interface. Therefore, the actual healing level of the asphalt mixture is lower than that of the interface. 

## 4. Conclusions

In this paper, the influence law of time and temperature on the self-healing index of the asphalt–aggregate bonding interface was measured, the influence degrees of aging, immersion, and other factors on the self-healing ability of interface strength were discussed, and the relationship between the self-healing degree of the asphalt–aggregate interface and the self-healing ability of the mixture was analyzed. Through this research, the following conclusions can be drawn.

A higher healing temperature and longer healing time can effectively improve the healing level of the asphalt–aggregate interface. 

The Compertz model can accurately describe the change in the healing index over time. Based on the time–temperature equivalence principle, the main healing curve of bonding strength can be established.

Immersion, salting solutions, and aging all have adverse effects on the healing properties of the bonding interface. The longer the immersion time, the worse the healing index of the interface. With the increase in solution concentration, the healing property of the interface between the asphalt and aggregate gradually decreases. With the extension of aging time, the self-healing ability of interface strength decreases linearly. There is a good correlation between common asphalt indexes and its healing ability.

The healing level of the asphalt–aggregate interface is linearly correlated with the healing level of the strength and fracture energy of the mixture. The actual healing level of the asphalt mixture is obviously lower than that of the interface.

It is feasible to simulate and analyze the self-healing of the asphalt mixture using the finite element method, which is not only green and sustainable, but also convenient. It should also be noted that more studies are required to verify the accuracy of the FE method. More experiments on various asphalt mixtures should also be conducted to ensure the robustness and accuracy of the model proposed in this paper.

## Figures and Tables

**Table 1:** Properties of AH-70# asphalt.

Items	Values	Test Methods
Penetration (25 °C, 0.1 mm)	75	ASTM D5
Softening point (°C)	47.8	ASTM D36
Ductility (15 °C, cm)	>100	ASTM D113
Flashing point (°C)	285	ASTM D92

**Table 2:** Properties of aggregates.

Sieve Size (mm)	16	13.2	9.5	4.75	2.36	1.18	0.6	0.3	0.15	0.075
Apparent gravity (g/cm^3^)	2.716	2.723	2.681	2.679	2.694	2.708	2.713	2.713	2.712	2.712
Water absorption (%)	0.49	0.57	0.63	0.52	-	-	-	-	-	-

**Table 3:** Elastic parameters for the aggregate and asphalt mortar.

Material	Elastic Modulus (MPa)	Poisson’s Ratio
Aggregate	20,000	0.2
Asphalt mortar	800	0.5

**Table 4:** Model parameters for cohesion before healing.

Interface Type	Fracture Strength (MPa)		Fracture Energy (J·m^2^)	
	Normal Direction	Tangent Direction	Normal Direction	Tangent Direction
Interface of aggregate–mortar	0.6	1	500	500
Interface of mortar	1	2	500	500

**Table 5:** Parameter table for Prony series viscoelastic coefficients of the asphalt mortar.

*i*	1	2	3	4	5
*g_i_*	0.542	0.166	0.1	0.098	0.034
*T_i_*	0.048	0.631	6.711	48.78	613.497

**Table 6:** Parameters of the *HI* model.

Parameters	10 °C	40 °C	60 °C	80 °C
*a*	1.000	1.000	1.000	1.000
*b*	−9.047	−10.140	−20.500	−12.050
*c*	−0.460	−0.792	−1.079	−0.994
SSE	0.003	0.006	0.003	0.000
*R* ^2^	0.969	0.976	0.991	1.000
RMSE	0.057	0.056	0.036	0.006

**Table 7:** $lg\alpha \prime_{T0}$ with 60 °C as the reference temperature.

$lg\alpha \prime_{T0}$	10 °C	40 °C	80 °C
Initial value	8.58	2.172	−1.457
Optimal value	6.65	1.80	−1.20

**Table 8:** $lg\alpha_{T}$ for different temperatures.

$lg\alpha_{T}$	10 °C	40 °C	60 °C	80 °C
Initial value	5.79	0.98	−0.78	−2.01
Optimal value	2.32	0.20	−0.08	−0.20
*R* ^2^	0.991	0.987	0.997	1.000
SSE	0.035	0.008	0.010	0.001

**Table 9:** Parameter values of the optimal *HI* principal curve model.

Parameters	*a*	*b*	*c*	*R* ^2^	Equation of Master Curve
Value	1.00	−11.70	−0.92	0.988	HI(t)=exp(−11.7exp(−0.92lgt/lgα))

**Table 10:** Model parameters for the CZM element at different healing levels.

Interface Type	Healing Index	Fracture Strength (MPa)		Fracture Energy (J·m^2^)	
		Normal Direction	Tangent Direction	Normal Direction	Tangent Direction
Interface of aggregate–mortar	0.8	0.48	0.8	400	400
	0.6	0.36	0.6	300	300
	0.4	0.24	0.4	200	200
Interface of asphalt mortar	0.8	0.8	1.6	400	400
	0.6	0.6	1.2	300	300
	0.4	0.4	0.8	200	200

## Data Availability

Not applicable.
